# Operation Note Transformation: The Application of Lean Six Sigma to Improve the Process of Documenting the Operation Note in a Private Hospital Setting

**DOI:** 10.3390/ijerph182212217

**Published:** 2021-11-21

**Authors:** Nicola Wolfe, Seán Paul Teeling, Marie Ward, Martin McNamara, Liby Koshy

**Affiliations:** 1 Beacon Hospital Beacon Court, Bracken Rd, Sandyford Business Park, Sandyford, D18 AK68 Dublin, Ireland; Liby.Koshy@BeaconHospital.ie; 2 UCD Centre for Interdisciplinary Research, Education & Innovation in Health Systems, School of Nursing, Midwifery & Health Systems UCD Health Sciences Centre, D04 V1W8 Dublin, Ireland or steeling@qmu.ac.uk (S.P.T.); martin.mcnamara@ucd.ie (M.M.); 3 Centre for Person-Centered Practice Research Division of Nursing, School of Health Sciences, Queen Margaret University Drive, Musselburgh EH21 6UU, UK; 4 Centre for Innovative Human Systems, School of Psychology, Trinity College, The University of Dublin, D02 PN40 Dublin, Ireland; marie.Ward@tcd.ie

**Keywords:** operation notes, patient safety, documentation, DMADV

## Abstract

Clinical documentation is a key safety and quality risk, particularly at transitions of care where there is a higher risk of information being miscommunicated or lost. A surgical operation note (ON) is an essential medicolegal document to ensure continuity of patient care between the surgical operating team and other colleagues, which should be completed immediately following surgery. Incomplete operating surgeon documentation of the ON, in a legible and timely manner, impacts the quality of information available to nurses to deliver post-operative care. In the project site, a private hospital in Dublin, Ireland, the accuracy of completion of the ON across all surgical specialties was 20%. This project sought to improve the accuracy, legibility, and completeness of the ON in the Operating Room. A multidisciplinary team of staff utilised the Lean Six Sigma (LSS) methodology, specifically the Define/Measure/Analyse/Design/Verify (DMADV) framework, to design a new digital process application for documenting the ON. Post-introduction of the new design, 100% of the ONs were completed digitally with a corresponding cost saving of EUR 10,000 annually. The time to complete the ON was reduced by 30% due to the designed digital platform and mandatory fields, ensuring 100% of the document is legible. As a result, this project significantly improved the quality and timely production of the ON within a digital solution. The success of the newly designed ON process demonstrates the effectiveness of the DMADV in establishing a co-designed, value-adding process for post-operative surgical notes.

## 1. Introduction

The key component of the patients’ medical record is their clinical documentation. It captures the patient care journey from admission to discharge, including diagnoses, treatment, and resources used during their care. [1,2]. When the documentation is complete, detailed, and accurate, it prevents ambiguity and improves communication between healthcare providers. Conversely, incomplete or inaccurate documentation can negatively affect the quality of patients’ care, resulting in delays, errors, longer lengths of stay (LOSs), and missed or incorrect post-discharge patient follow-ups, ultimately leading to higher readmission rates and increased costs [1,2]. The Operation Note (often termed the “op note”) is an essential document that records exactly what surgical operation a patient had, key findings during surgery, and what the post-operative instructions for further patient care from the surgeon are for colleagues, and immediately post-op, for the Post-Anaesthesia Care Unit (PACU) staff. Therefore, it is a key document to inform patient care throughout the post-operative procedure period [1,2].

In Ireland, the Health Service Executive (HSE) does not have a specific document outlining the requirements for the ON. It is referenced within the document entitled “Standards and Recommended Practices for Healthcare Records Management” 2012 [3]. However, this relates primarily to the physical condition and maintenance of patient notes. Therefore, The Royal College of Surgeons of England (RCS) guidelines produced in 2014 are considered the gold standard for operative notes. They identify that “effective written communication should be accurate, clear, legible, comprehensive, and contemporaneous” [3].

This project was set in a private hospital in South Dublin. The hospital’s partnership with the University College Dublin (UCD) has led to over 200 staff trained in Lean Six Sigma (LSS) since 2017, who lead and/or contribute to the hospital’s ongoing system-wide quality improvement projects. The university program has been instrumental in the development of an LSS culture of improvement and, as part of the ongoing LLS work, an improvement project was undertaken in 2020 within the operating room (OR).

Lean Six Sigma (LSS) is the merger of two methods used in the quality improvement of processes. Lean developed in Toyota factories focuses on the elimination of non-added activities [4]. Six Sigma was introduced by Motorola to optimise its manufacturing processes by reducing their variability through the rigorous application of process metrics collection and statistical analysis [5]. Since the early 2000s, LSS thinking has been adapted into healthcare with the goal to improve patient safety, quality of care, efficiency, patient satisfaction, and performance [6,7,8,9,10,11,12,13,14]. LSS puts an emphasis on working with and eliciting the customers’ voice [15] and has been shown to be synergistic with person-centred approaches to engagement [14]. It was therefore seen as an approach that could both improve the existing process for ON documenting and engaging our primary end-users, the surgeons who did so.

**Figure 1:**
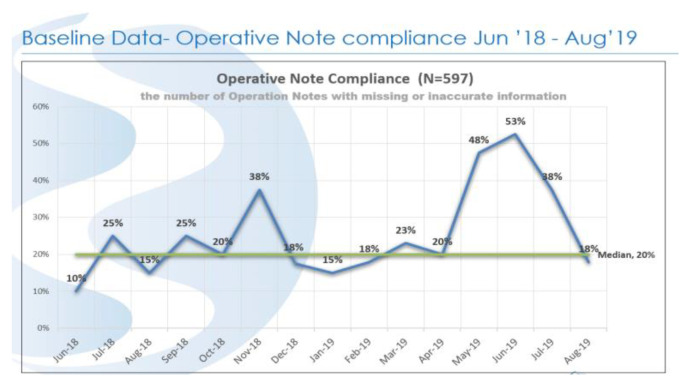
The number of ONs with missing or inaccurate information.

There are nine ORs that perform a range of elective surgeries such as orthopaedic, cardiac, cancer, and robotics. The ON average OR capacity is approximately 90% per day; therefore, the turnaround time is critical. Turnaround time is defined as the gap between application of the final dressing and the knife to skin on the next patient [15]. Any delays in the OR can lead to increased hospital costs, as well as patient and staff dissatisfaction [16,17,18]. An individual OR suite can have up to 15 procedures on an OR suite schedule, so the time between cases is critical, and there is an emphasis on what is termed “turnover” time between cases. The pressure of this quick pace can, however, impact the surgeon’s attention to the completeness or accuracy of the ON. A review was carried out in 2018/2019 to measure compliance in completeness and accuracy of the ON, and it was found that between June 2018 and August 2019, the median range was 20% (Figure 1).

The median of 20% compliance in completeness and accuracy of the ON was due to the following issues:

- Incomplete: notes were not completed in full and 80% had missing information;
- Vague: 27% of notes were lacking detail and difficult to understand;
- Abbreviations: 4% of notes included unapproved abbreviations;
- Illegible: in 3% of cases, handwriting was difficult to read.

There is a common phrase used by healthcare professionals “if it is not written down, it did not happen”, and in the same vain, “if is not readable, it is not complete” [19]. This means that if there is no record of the care that was delivered in the patient’s chart, by way of complete and accurate documentation, the activity is considered as not having been carried out. Poor documentation is a key safety and quality risk, particularly at transitions of care where there is a higher risk of information being miscommunicated or lost [20]. This can potentially lead to:

- Delays in care;
- Misinterpretation of care instruction;
- Contributions to inaccurate quality and care information;
- Compromising of safe patient care.

The study site of this project was accredited through Joint Commission International (JCI) and, as part of the hospital’s ongoing internal audit, the ONs were highlighted as an area of potential risk leading the hospital to deploy a Strategic Improvement Plan to improve compliance. Technology was seen to offer a viable solution to these issues, and the hospital’s existing Information System MEDITECH was suggested as a viable platform for ON documentation. This was the basis for the introduction of a digital ON instead of the manual paperwork in use up to this point in time. However, one year after the introduction of MEDITECH, only 11% of surgeons (*n* = 96) were documenting using this technology. This reflects the experience in process improvement that change will only occur if staff recognise the need for change [21]. The team could see that a new more person-centred approach was needed, and as the hospital had experience of and success with LSS for improvement, it was decided to utilise the LSS methodology to review the process for ON completion and then redesign/design as appropriate.

## 2. Methodology

The project was adopted to improve the accuracy and completeness of the ON. The framework adopted in this project was initially DMAIC (Define-Measure-Analyse-Improve-Control); however, it was quickly understood that the best improvement would result from utilising a Design for Six Sigma, DMADV (Define-Measure-Analyse-Design-Verify) approach. DMAIC measures the current performance of a process, while DMADV measures customer specifications and needs (Table 1) [22].

### 2.1. Define Phase

The steps of DMADV furnish a model for a structured approach initialised in a project overview document known as a Project Charter with the model allowing for evaluation and re-evaluation of the project outcomes [24]. The LSS tools utilised within this project are outlined in Table 2. A charter was prepared to define the problem, the scope of the project, its goals, and any potential risks associated with the project implementation. This was signed off by the project sponsors, the Chief Information Officer and Chief Operations Officer, with the aim of designing a digital system that would facilitate the following goals:

- 100% of all ON to be completed digitally;
- 100% of all mandatory fields identified by JCI and RCS to be completed;
- 100% of surgeon design and functionality requirements met;
- Automated auditing through an existing BI (Business Intelligence tool).

All surgeons working with the study site and all surgical procedures were included in the scope of this project. A multidisciplinary team (MDT) was formed to deliver the project collaboration with and inclusion of all the relevant stakeholders. The MDT consisted of representatives from the quality department, nurses (from PACU, theatre, and anaesthesia), and members of the IT departments. To represent the surgeons, the heads of each of the surgical services departments were included as part of the MDT; this represented 9.6% of surgeons working in the hospital (*n* = 96). A communication plan was developed and monthly stakeholder engagement sessions were held to stimulate discussion and debate and to encourage the team to share their feedback on the existing MEDITECH digital system and how it currently met their specific requirements and preferences.

LSS has a strong emphasis on eliciting and acting on the “Voice of the Customer” (VOC) and understanding customer expectations of service [25]. VOC feedback from the entire team on the current MEDITECH digital system included:

- “It’s not user friendly. I can’t use it where I want to and it won’t work on my iPad”;
- “It doesn’t even have the basic functionality of spell check and I need to be able to draw! I can’t amend documents”;
- “I can’t read the doctors note and end up going around the house trying to figure out what happened in theatre”;
- “It’s ridiculous that I have to fill in the same fields when the information is already on the system”;
- “I don’t know what you want me to fill in!?”;
- “Auditing Op notes is painful. Trying to decipher the scribbles on the page to see if it meets the standards is impossible and doesn’t work because you don’t get a true reflection of a surgeons work”.

Initial analysis from these meetings found that the current MEDTECH system did not meet the functional requirements of the main stakeholder, i.e., the surgeons. It became clear that stakeholders had different views of what constituted value-added (VA) and nonvalue activity (NVA). NVA refers to any work activity that consumes resources but does not add value or contribute to the “customer”. Value-added (VA) refers to any work activity that contributes in a meaningful way to the process [26].

**Figure 2:**
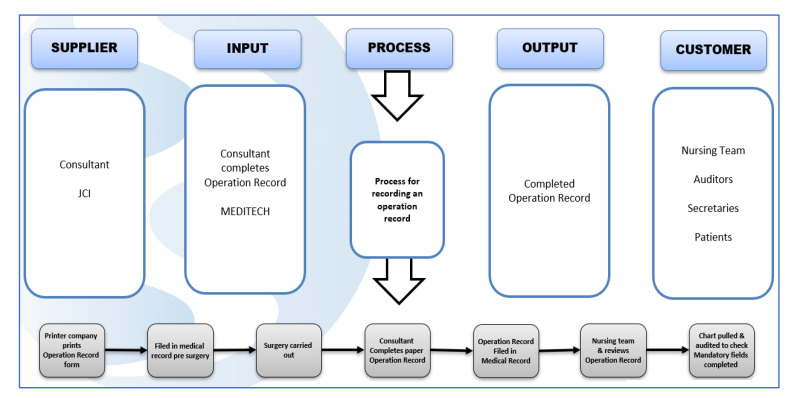
SIPOC.

Following this initial VOC, we worked with stakeholders to gain an understanding of the process. This was facilitated by the use of a SIPOC tool (Suppliers, Inputs, Process, Outputs, and Customers), which is shown in Figure 2. The SIPOC allows a high-level overview of the process [27]. In this case, the process for ON completion and also facilitating the stakeholder’s discussion on stakeholder engagement required the initiation and sustaining of any required change in practice.

Having completed a SIPOC and our initial customer voice, the MDT then proceeded to identify areas of the process to measure.

### 2.2. Measure Phase

**Figure 3:**
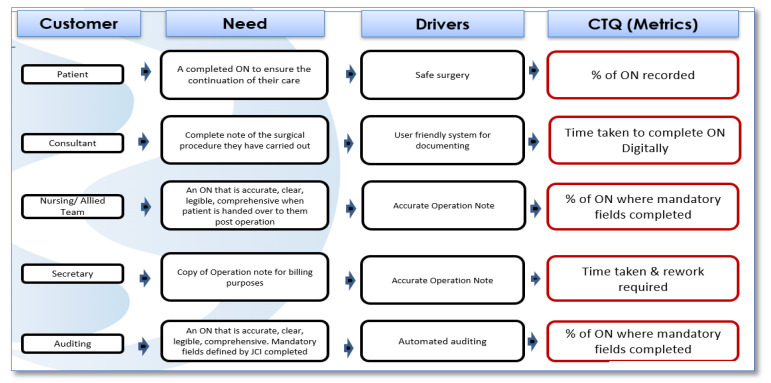
CTQ.

Within the measure phase, the voice and identified needs of the stakeholder (customer) from our extensive stakeholder engagement were mapped to CTQ (Critical to Quality) templates (Figure 3), enabling us to develop metrics that captured the customer voice and guide the development of a new process. A CTQ essentially identified the issues or factors that were critical to the customers and put them into specific, actionable, and measurable performance requirements [28]. The key measures identified for collection were:

1. Percentage of ON compliance in completing mandatory fields;
2. Time taken to complete an ON.

**Figure 4:**
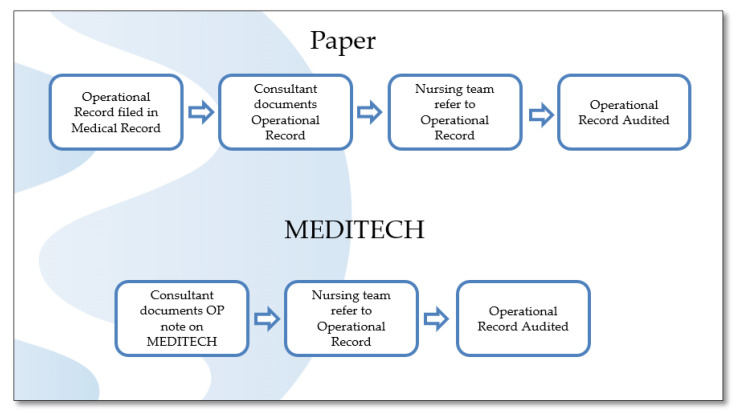
As we think it is process map.

**Figure 5:**
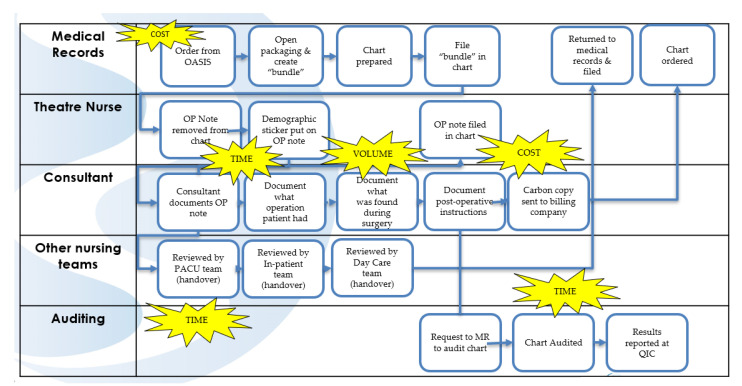
“As it is” process map.

To visualise the process at a more detailed level than the SIPOC, we undertook extensive process mapping [28]. The first step was to map the “as we think it is” process map (Figure 4) to create a shared understanding of the process and how it should work. We then carried out a Gemba, which is Japanese term meaning “the actual place”. A Gemba is the action of going to see the actual process, understand the work, ask questions, and learn (Figure 5). This allowed the team to capture what was happening within the system (“as it is” process map) and identify the NVA for elimination. This then provided a benchmark against which to measure future improvements [28].

The team identified the mandatory field requirement in line with RCS and JCI guidance:

- Patient Name;
- Patient Date of Birth;
- Visit number;
- Date of surgery;
- Surgeons Name;
- Anaesthetists name;
- Surgical Assistants name;
- Theatre number;
- Procedure code;
- Surgical description;
- Estimated blood loss;
- Post-surgery diagnosis;
- Complications;
- Post-surgery instructions;
- Surgeons signature;
- Surgeons Irish Medical Council (IMC) number;
- Date and time the ON completed.

The “as it is” process map (Figure 5) assisted in planning for data collection. Data collection primarily consisted of Gemba and audit. The Gemba exercise was carried out over a two-week period at varying times of the day to ensure the wide capture of surgeons and specialties. To confirm that a bias did not enter into the mapping process, the data and observations gathered were verified with the nurses, technicians, support staff, and surgeons via individual interviews and meetings, and they validated the findings. The Gemba and audit informed an “as it is process map” (Figure 5) often known as a “current state” process map, which corroborated the areas of NVA identified in the VOC sessions.

### 2.3. Analyse Phase

Applying Lean Six Sigma in healthcare settings has proven beneficial in mapping the patient journey to identify NVA and to improve care, effectively by making the invisible NVA visible [29]. Stakeholders expressed that the current state process map reflected what they intuitively knew, that there were issues with the process, but it was not until they saw this visualised on the current state map that they realised the extent of these problems. To identify the waste in the process, we used the LSS acronym “TIMWOODS”, which is shown in yellow in Figure 5 and in detail in Table 3 [30].

## 3. Time Taken to Complete the ON

Our findings indicated that the process for completing an ON can take anywhere from 1 min to 55 min, with an average completion time of 10 min (*n* = 25). The digital process that had been introduced was intended to be a faster process than the manual ON it had replaced; however, it actually took surgeons longer to complete. A control test was carried out by a MEDITECH expert who was timed in completing an ON, and they completed the process in 20 min digitally. It was found that just to gain access to the digital form, there were eight clicks on the screen. In addition, the digital form had almost double the number of fields to be completed compared to the hardcopy form. The use of MEDITECH to document the ON Notes was, in essence, applying a technical solution rather than an adaptive long-term change [31].

## 4. Incomplete ON

**Figure 6:**
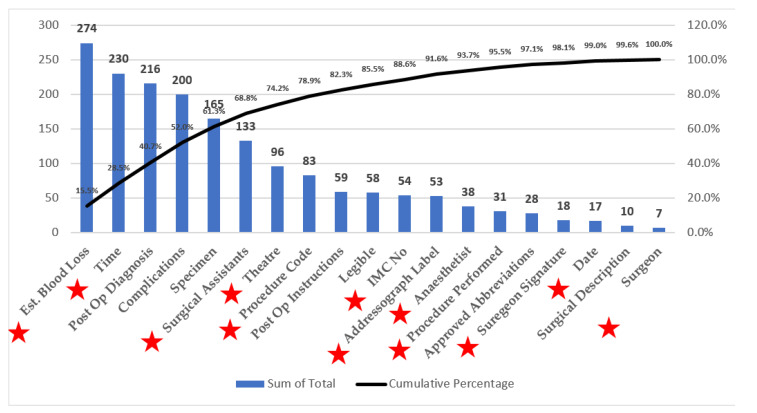
Pareto analysis of ON compliance and the number of fields already captured (*n* = 1770).

Pareto Analysis (Figure 6) was carried out on the incomplete fields on the ON (*n* = 19), and it was found that 63% of the fields not completed were actually captured elsewhere within MEDITECH by the nursing team; for example, blood loss, time of surgery, surgical assistants, procedure code, theatre number, and date were all classed as missing information even though they were captured elsewhere.

## 5. Rework

No incidents of rework (carrying out the same process steps again) were observed during the Gemba, due to illegible or missing handover information. To obtain this information, a questionnaire was administered to a purposive sample of 10 members of the PACU nursing team (48% of PACU) to ascertain the volume of questions they had to make to surgeons based on the legibility of the ON. The PACU nursing staff stated:

- “Chasing the surgeons around theatre”;
- “Having to phone the surgeons at their homes to clarify details”;
- “One time I had to sit in the restaurant while the surgeon filled in the Op Note”.

We provided a frequency scale to the PACU nurses (*n* = 10) to identify how often they had to follow up queries due to illegible writing or unclear instruction, and 70% of nurses said it occurred “often”.

**Figure 7:**
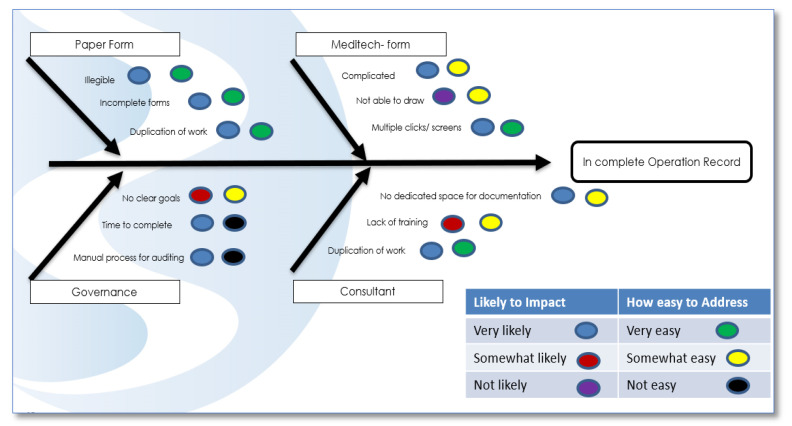
Ishikawa (fishbone) diagram.

The project team used the LSS tool, an Ishikawa (fishbone) diagram (Figure 7), to brainstorm with the stakeholders to discover the root cause of the problems identified [29], and a Failure Mode Effect Analysis (FMEA) was completed to highlight the main risk areas. The Ishikawa diagram was used to map our collected data and Gemba results to visually indicate the root causes leading to the incomplete operation record using the MEDITECH digital system. We mapped these to see the likely impact of these root causes on the process of ON completion and the frequency of their occurrence.

### 5.1. Design Phase

The result of Gemba was presented to the stakeholders and a brainstorming session was held to identify potential improvements. However, despite reviewing all of the collected and analysed data, stakeholders felt that they had reached an impasse and did not know how to process. It was agreed that the project team would take this data to the in-house IT development team (design team) and feedback to our stakeholders. LSS is useful in challenging the thinking of “the way we’ve always done it” [32], “that’s not how we do things here” [33], or “we’ve tried it before and it didn’t work” [34]. Using this approach, we posed the question “If our hands no longer tied by constraints of the MEDITECH system, how would you build an ON?”

**Figure 8:**
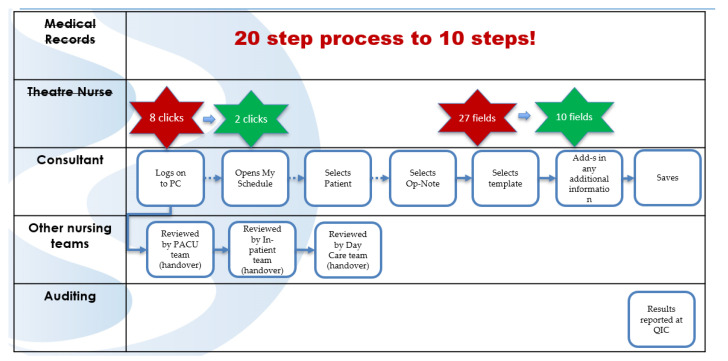
“As it should be” process map.

The design team then proceeded to examine this process in more detail. To do this, they worked together to identify the step-by-step nature of the process and documented a “future state” or “as it should be” process map (Figure 8).

The list of problems with the MEDITECH system was flipped into a functionality list of requirements for a new system (Table 4), informed by our stakeholders’ VOC sessions, and it was decided to build a “demo” version of the application. The “demo” version was built to be shown to the key stakeholders, to convince them to try it out and sign off that it would meet their needs before the IT team spent weeks formatting and building complex databases that may not be used.

The project team completed a Failure Mode Effect Analysis (FMEA) to highlight the potential main risk areas [29]. The FMEA enabled us to assess the potential ways in which each sub-process might fail, and these failure points were then analysed in terms of effects and causes. A risk priority number (RPN) was assigned to each potential failure point, depending on its severity, frequency, and detectability. The highest risk was identified and then incorporated into the design of the application. For example, the highest risk identified was incomplete fields, and to ensure that this could not occur, controls were put in place when designing the application so that these fields were mandatory and the ON could not be submitted unless completed.

**Figure 9:**
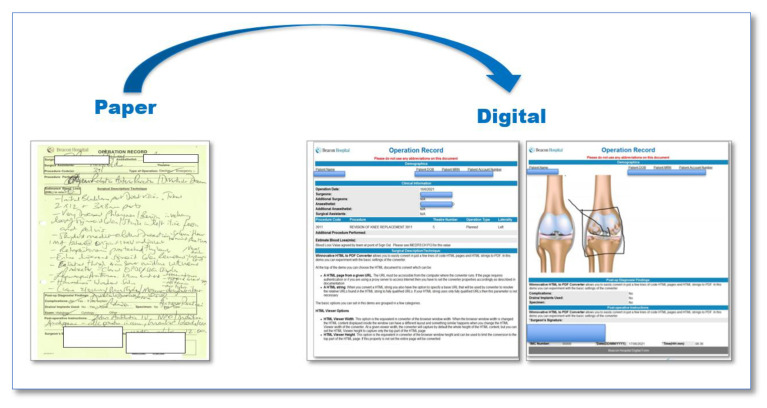
The newly designed form.

A previous LSS study within the hospital relating to patient scheduling developed an application called “My-Schedule” that provides surgeons with a worklist of patients in easy webpage format on the hospital intranet, and it was decided to build on to this work. Effectively, it means that from a worklist of their own assigned patients, the surgeons can select the “Digital Documents” button, which launches a webpage version of the ON. This application is able to pull 63% of the information from Meditech and allows the surgeons to build templates for their procedure codes to speed up the typing process. We utilised two tools to inform the design of the new digital form (Figure 9):

- The ISBAR framework: This represents a standardised approach to communication and is a stand utilised across the hospital for all forms of communication. It stands for Introduction, Situation, Background, Assessment, and Recommendation [35].
- 5S: This is a 5 step Lean methodology: Sort, Set in Order, Shine, Standardise, and Sustain. We applied the 5S principles to declutter the paper form to ensure the form was clean and uncluttered [36].

### 5.2. Verify Phase

**Figure 10:**
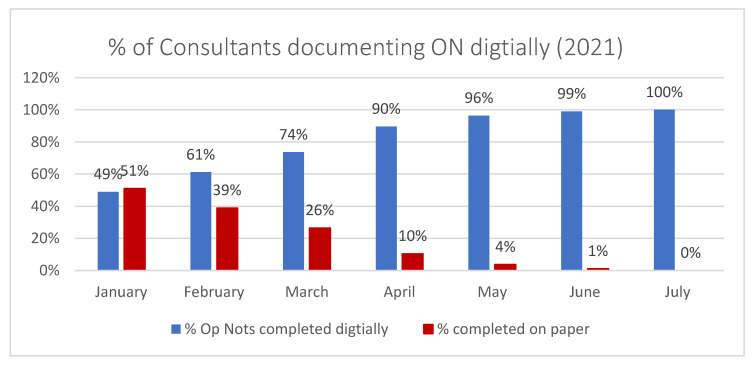
% of consultant documenting ON digitally.

To verify that the new application met the customer’s needs, an evaluation plan was put in place to monitor bugs and issues with the system. This was overseen by the Head of Surgical Services. An initial evaluation was carried out 6 weeks post-go live with another at 6 months post-go live to ensure it is performing to expectations. The hospital has a business intelligence tool that presents historical data in a visual “dashboard” to allow end users to monitor compliance and make data-driven decisions. A dashboard was created for ON monitoring (Figure 10).

## 6. Results

On completion of this project, 100% of all surgeons were and are documenting the digitally utilisation of the new application. Figure 10 shows the impact of the measures introduced into the system and how, through continuous deployment of the application across the Surgical Services department, the targets have been met.

A total of 63% of the data on the ON were already captured by the nursing teams on MEDITECH, and the application was designed to auto-populate this information onto the new form. The application also allowed for the surgeons to create templates for different surgeries. When documenting, the surgeons can select the specific template, and it populates with pre-formatted details of the surgery without having to start from scratch every time. From utilising these features in the new application, there has been a reduction of 30% in time to compete the ON (*n* = 17)

As a result of this improvement, costs have reduced from EUR 10,000 annually in printing and transport of the paper Operative Notes. Costs are also expected to reduce in the form of direct labour required, as well as time and effort spent undergoing non-value-added tasks such repeated filing in the medical record.

The nursing team in PACU (Post-Anaesthesia Care Unit) no longer have to “chase” the surgeons for clarification and there is a smoother process flow within the department without interruption. VOC example: *“I love it! It is saving me and my team so much time, finally no more chasing to try and get the details we need. No more following up with queries, its brilliant!*

The surgeons are also extremely satisfied with the end product. Specifically, 100% of their functionality requirements have been met. Examples of surgeons’ VOC:*“Easy to use”, “Love the layout”, “Excellent system you should patent it!”, “Very happy everything is great”.*

All required fields identified to comply with JCI and RCS standards have been met. These fields are mandatory and can no longer be incomplete, i.e., if not complete, the ON cannot be saved.

From an operational perspective, as the new ON has eliminated handwriting and is typed, it is 100% legible. The feedback from the PACU nursing team reading is extremely positive. VOC example: *“the novelty of actually being able to read the handwriting and understand the detail of the surgery is brilliant!”*

From an auditing perspective, the data are automated into both the hospital patient tracker system and hospital’s Business Intelligence Tool. The team can have real-time visualisation of the Operative Note, and can see that it has been completed before the transfer of care from the surgeon and anaesthetist to the PACU nursing team, ensuring the change is sustained.

## 7. Discussion

This project aimed to explore methods to overcome barriers to the implementation of a paper to digital solution. The project was person-centred in its engagement, and clinician and clinical staff engagement throughout the project were a key factor in successful implementation. Input from the entire team not only allowed for better issue identification and solution generation, but also increased team cohesiveness and motivation to actively participate in the project [37]. As with any change management project, the project team encountered some problems with staff who were apprehensive about the change. Examples of barriers that the project team experienced were a lack of commitment from operational staff and a misunderstanding of the timeframe to develop and deploy the application. In hindsight, they were responding to their condition within the system. As customers, they were feeling righteously “done to” (i.e., screwed) and were reacting to the “dance of the blind reflexes” [38]. Instead of giving up when the clinical team stated “I don’t know what I want but I know it’s not this” on reflection, this should have been anticipated and, for future projects, customers should be encouraged early as a partner, not late as a judge. The project has helped facilitate the development of a continuous improvement culture not only in the Surgical Service Department but also more broadly in our Healthcare organisation, with the lessons learnt in this study now being applied to other papers to digital solutions in the hospital.

Enablers of this project included a system-wide deployment of LSS within the hospital and the high number of LSS practitioners actively working in the hospital. The hospital also has an education and training academy partnered with UCD that oversees LSS deployment. Dickson et al. (2008) suggested that healthcare staff relate to LSS better if working with other healthcare staff who are LSS practitioners, rather than the usual overreliance on industry consultants [39]. Jones (2017) similarly suggested that staff developments of Lean Six Sigma skills are best “nurtured and sustained” by other staff members/colleagues acting as mentors or coaches [40]. The fact that this project was led and resourced by an internal LSS multidisciplinary team was therefore seen as an enabler for change.

A limitation to this study could be that the project did not formally measure the impact of the digital solution on the time between cases in the Surgical Services department and how it has improved the continuation of care along with patient flow. Pre- and post-implementation digitalisation would have better clarified the efficacy of the solution. However, this feedback has been fed forward to the project team and the Education and Training Academy and will feed-forward into future system design and the next phase of this project. There are, however, many strengths to this study, most notably the use of DMADV over DMAIC. Studies have found that 70–80% of problems are centred on the design of a product [41]. The DMADV methodology enabled the team to create a high-quality product while keeping the customer’s wants and needs in mind during each phase.

The project has facilitated improvement in the accuracy and completeness of the ON whilst also impacting positively on the operations of the Surgical Services department. The LSS methodology was instrumental in the successful rollout, assessment, and sustainability of this project. It adds to the growing body of literature demonstrating that LSS can be used in healthcare settings to improve system efficiency and reduced “waste”.

## 8. Conclusions

The hospital had attempted to improve compliance in documentation of the Operative Notes for several years with no significant difference made. This project shows that the application of LSS and, in particular, DMADV can be used to structure product development. Significant improvements were observed in the key performance metrics after the implementation of the LSS strategy. LSS succeeded in a process where previous improvement attempts had failed. This is attributed to the structured data collection that focused attention on the true causes of the problem.

The success of this project has encouraged stakeholders who were initially hesitant to engage in the project to become champions for change. Several suggestions have been put forward for potential future enhancements and to disseminate across the hospital. As the application of the successful Lean Six Sigma is increasing across the hospital, it contributes to the continuous development of the Lean culture within the organisation, and the lessons learned can be applied in other healthcare institutions.

## Figures and Tables

**Table 1:** Difference between DMAIC and DMADV [23].

DMAIC	DMADV
The emphasis of DMAIC is more on correcting an existing process and reducing existing variation	DMADV is more about creating a process (standardise) with an optimised design or “doing it right the first time”
DMAIC is “correction”	DMADV is prevention
DMAIC uses more of a statistical tool and numerical/quantitative analysis to arrive at the solution	DMADV uses qualitative tools: QFD (Quality function deployment), KANO model, etc.
Six Sigma focuses on one of two CTQs, looks at processes, and aims to improve the CTQ performance	Design for six sigma focuses on every single CTQ that matters, looks at products and services, as well as the processes by which they are delivered, and aims to bring a large-scale improvement
DMAIC projects often take a short duration to fix a customer problem and process improvement	DMADV projects are often much larger, take longer, and are often based on a long-term business need for a new product or service

**Table 2:** The Lean Six Sigma Tools utilised in the project.

Improvement Tool	Description of Tool
Project Charter	A Project Charter was used to define, act on, and review challenges and problems
SIPOC (Figure 2)	The SIPOC was used to provide a high-level view of the process. SIPOC stands for Suppliers, Inputs, Processes, Outputs, and Customers
CTQ (Figure 3)	CTQ stands for Critical to Quality tree and was used to capture the key measurable characteristics of the process that must be met in order to satisfy the customer
Process Map (Figures 4, 5 and 8)	Visually shows the individual steps within a process
Pareto Analysis (Figure 6)	A Pareto chart is a bar chart that arranges the bars (counts) from largest to smallest, from left to right. Helps by visually identifying the most frequent defects
VOC	Voice Of Customer: Allowing the customer voice to be heard to pull from the process
Gemba	Observation/understanding of where and how the work is carried out
Ishikawa diagram (Figure 7)	Additionally known as a fishbone diagram. It combines brainstorming and mind mapping to discover the cause-and-effect relationship of an underlying problem
5S’s	Five steps of this methodology: Sort, Set in Order, Shine, Standardise, and Sustain. 5S create a clean, uncluttered environment
FMEA	FMEA stands for Failure Mode and Effect Analysis. It is a toll used to analyse risk to prevent an event happening. It highlights the aspects of a process that should be targeted for improvement
TIMWOODS (Table 3)	Acronym of transportation, inventory, movement, waiting, overprocessing, overproduction, defects, and skills. Facilitates the identification and classification of the types of waste

**Table 3:** Waste Analysis—TIMWOODS.

	Waste	Impact	Identified
T	Transport	Moving items or information	Shipping hard copies
I	Inventory	Items or information that customer has not received	Bulk buy Op notes from printing company
M	Motion	Excessive movement within workspace	Repeated filing in Medical Records-Opening tabs
W	Waiting times	Waiting for information or items to arrive	Waiting for ON to be completed before transfer Poor technical design
O	Over-Processing	Doing more work than necessary	63% of fields populated on paper form are already on MEDITECH
O	Over-Production	Doing work before it is needed	MEDITECH duplicate entries and entering past fields
D	Defects	Mistakes and errors that need to be reworked	Illegible forms Mandatory fields not completed
S	Skills	Not using workers’ greatest abilities	Not utilising the skills of the consultant, wasting time filling in a form

NVA identified via Gemba of the “As we think it is” Process Map.

**Table 4:** Requirements list.

End User Functionality Requirement	How This Was Incorporated into the Build
Fast, easy access, and user-friendly (web design)	Built on a webpage. Can be accessed through the internet (page already in use across hospital). Only one click to access OP Note
Auto-populate any existing data already captured in MEDITECH	HL7 interface with MEDITECH to pull data
Can be used on any device and accessed anytime anywhere	On intranet, can be accessed using any device through remote working station.
Ability to build templates	Surgeons have the ability to create, edit, and delete as many templates as they wish
Ability to draw	Surgeons can draw using stylus pen on a tablet in theatre
Amendment functionality	Surgeons have the ability to amend documents if required whilst still maintaining the original details
